# The Price of Coal (Limitation) Act, 1915

**Published:** 1915-08-14

**Authors:** 


### THE PRICE OF COAL (LIMITATION) ACT, 1915.

### A Hospital Superintendent's Plea for Action.

The attitude of the British Medical Association in preventing hospitals from obtaining the concession originally offered by the Chancellor of the Exchequer for the repayment of the surtax on rectified spirit, which attitude is largely responsible for the indefinite position in which the voluntary hospitals now stand in having the repayment of surtax approved in principle but not embodied in an Act of Parliament (see The Hospital of July 24, p. 343), shows only too plainly the necessity to secure a representative in the House of Commons who is in close touch with the work and needs of the hospitals. He would undertake the responsibility of keeping the views of hospital administrators and the position of the hospitals before the Legislature. The attitude of the British Medical Association is also, we fear, the attitude of many members of Parliament, who realise in principle the great work of the voluntary institutions, but do not see their way to help to secure for them concessions which may justifiably be applied for, for they would be granted if backed with earnestness and enthusiasm on the part of a few members.

There is, however, one feature evidenced by the present position, which has shown administrators of hospitals that always they must be on the look-out to safeguard the interests of their hospitals, and to insist upon the beneficent work our voluntary hospitals do for all classes. By such a course only will the voluntary hospitals obtain justice and adequate recognition.

The Price of Coal (Limitation) Bill recently submitted to Parliament calls for the careful consideration of hospital administrators and the British Hospitals Association, if this body could only make itself the recognised pulsimeter of the hospitals' life and work. The Association at present lacks initiative and the services of a few capable and proved organisers. Our readers will recall that this Act seeks to limit the price of coal at the pit's mouth to a standard advance of not more than 4s. per ton on the corresponding date of last year, "or as near thereto as having regard to the course of business may be practicable." The purpose of the Act is thus to ensure that the consumer of coal shall not be called upon to pay a fictitious increase in price by reason of war conditions. It will be remembered that the price of coal in London last winter rose to an average certainly not below 9s. a ton over the corresponding price of the previous year, and Mr. Runciman, in introducing the Bill, intimated that if this increase was general throughout the country it represented a rise in price of no less than £9,000,000.

On the face of it this Act, as passed, would appear to be altogether to the advantage of the whole community; but there are several provisions in the Bill which need very careful consideration.

Clause 4 (2) provides that "This Act shall not apply to the sale of coal supplied in pursuance of a contract made before the commencement of the Act," and that "where any contract has been made on and after April 1, 1915, and before the commencement of this Act for the sale of coal by the owner thereof at the pit's mouth, coal delivered under that contract, after the expiration of the period fixed under this provision and *shown to be excepted coal* within the meaning of this provision, shall, if the other party to the contract within two months after the commencement of this Act gives notice in writing to that effect to the owner of the coal at the pit's mouth, be deemed for the purposes of this Act to be sold at the time of the delivery thereof."

"If, in consequence of this provision, the price to be paid by any person to whom coal is delivered is reduced by any amount, the price to be paid by any person to whom the coal is delivered in pursuance of any subsidiary contract shall be reduced by an equivalent amount; and any purchaser under any such subsidiary contract shall have the same right to give notice to the owner of the coal at the pit's mouth as the person who has made the original contract with that owner, and any person who has sold the coal shall, if required, communicate to the purchaser the name of the person from whom the coal has been bought."

"For the purposes of this provision 'excepted coal' means coal supplied for domestic or household purposes to any person, and coal supplied for any purpose to any local authority, or to any undertakers supplying gas, water, or electricity in any locality in pursuance of authority given by an Act of Parliament or by an Order confirmed by, or having the effect of, an Act."

"(3) This Act shall not apply to coal raised in Ireland."

"(5) This Act shall have effect during the continuance of the present war, and a period of six months thereafter."

Two clauses of the Act quoted above have very definite bearing upon hospital work, and, therefore, deserve the careful attention of hospital administrators:—

(*a*) Where "excepted coal" is supplied under a contract made on or after April 1, 1915, and before the commencement of the Act, power is given to the purchaser under the contract to give notice to the owner of the coal at the pit's mouth [to adopt the Act], and the coal shall then be deemed for the purposes of the Act to be sold at the price and time of delivery.

This clause would appear to give the purchaser of "excepted coal" the power to determine an existing contract should he be paying more under contract than the standard increase of 4s. per ton on the price for the corresponding period of last year, and to buy at the market price of the day.

(*b*) . . . The coal, however, must be shown to be "excepted coal"—Clause 4 (2)—that is, "coal supplied for domestic or household purposes to any person, and coal supplied for any purpose to any local authority," etc.

It will be observed that this latter clause, if it does not expressly exclude *trade or manufacturing* purposes, excludes them in effect, and it therefore behoves hospital administrators to see that no limitation of words, as in the recent unfortunate experience in connection with the promised refund of surtax on rectified spirits, "*subject to the agreement of all parties*"—a limitation that at once rendered the concession inoperative—deprives the institutions of any privilege or concession which the Price of Coal (Limitation) Act should offer to them. One notes that Mr. Runciman, in introducing the Bill, definitely stated, "it had been suggested that the Bill should be limited only to household coal. That would be unfair not only to householders who were dependent on manufacturing industries, but unfair also to those industries and to the State, which had now a large interest in keeping down the working expenses of the railways, etc. (*Times*, July 20). "Another criticism of the Bill," he proceeded, "was that it would not apply to merchants. He wished they could make it apply to the middleman, but he had been unable to devise any means whereby they could get over the middleman's prices and profits, except by taxation."

The very complexity of the interests involved referred to by Mr. Runciman may not unlikely have the effect of causing us to see "lions in the path," but it will be noticed that Mr. Runciman's definite statement with regard to the inclusion in "excepted coal" of coal supplied for manufacturing or trade purposes, is not embodied in the Act, and it is quite clear that unless an Order under the Act is promptly obtained manufacturers, and, indeed, all business concerns, may be excluded from the enjoyment of the standard price on which the Act is based. Hospitals must, therefore, see to it that the voluntary institutions are included *by Order* in the "excepted coal" provision clause. They have got "so near" and, thanks to the British Medical Association, found themselves "yet so far" before, that if they do not look ahead they may find their position once more destroyed. We hoped the words "to institutions" would have been inserted in Clause 4, line 27, after the words "to any local authority"; the clause would then have read:—

"For the purpose of this provision 'excepted coal' means coal supplied for domestic or household purposes to any person, and coal supplied for any purpose to any local authority, to institutions, or to any undertakers supplying gas, water, or electricity," etc.

The same results for hospitals can yet be secured by an Order, but there is no time to lose in securing this. We shall welcome the observations of hospital administrators on the omission in the Bill in view of its importance to voluntary institutions.

